# Gene expression in tumor cells and stroma in dsRed 4T1 tumors in eGFP-expressing mice with and without enhanced oxygenation

**DOI:** 10.1186/1471-2407-12-21

**Published:** 2012-01-17

**Authors:** Ingrid Moen, Charlotte Jevne, Jian Wang, Karl-Henning Kalland, Martha Chekenya, Lars A Akslen, Linda Sleire, Per Ø Enger, Rolf K Reed, Anne M Øyan, Linda EB Stuhr

**Affiliations:** 1 Department of Biomedicine, University of Bergen, Jonas Lies vei 91, 5009 Bergen, Norway; 2 The Gade Institute, University of Bergen, Bergen, Norway; 3 Department of Microbiology, Haukeland University Hospital, Bergen, Norway; 4 Department of Pathology, Haukeland University Hospital, Bergen, Norway

## Abstract

**Background:**

The tumor microenvironment is pivotal in tumor progression. Thus, we aimed to develop a mammary tumor model to elucidate molecular characteristics in the stroma versus the tumor cell compartment by global gene expression. Secondly, since tumor hypoxia influences several aspects of tumor pathophysiology, we hypothesized that hyperoxia might have an inhibitory effect on tumor growth *per se*. Finally, we aimed to identify differences in gene expression and key molecular mechanisms, both in the native state and following treatment.

**Methods:**

4T1 dsRed breast cancer cells were injected into eGFP expressing NOD/SCID mice. Group 1 was exposed to 3 intermittent HBO treatments (Day 1, 4 and 7), Group 2 to 7 daily HBO treatments (both 2.5bar, 100% O_2_, à 90 min), whereas the controls were exposed to a normal atmosphere. Tumor growth, histology, vascularisation, cell proliferation, cell death and metastasis were assessed. Fluorescence-activated cell sorting was used to separate tumor cells from stromal cells prior to gene expression analysis.

**Results:**

The purity of sorted cells was verified by fluorescence microscopy. Gene expression profiling demonstrated that highly expressed genes in the untreated tumor stroma included constituents of the extracellular matrix and matrix metalloproteinases. Tumor growth was significantly inhibited by HBO, and the MAPK pathway was found to be significantly reduced. Immunohistochemistry indicated a significantly reduced microvessel density after intermittent HBO, whereas daily HBO did not show a similar effect. The anti-angiogenic response was reflected in the expression trends of angiogenic factors.

**Conclusions:**

The present *in vivo *mammary tumor model enabled us to separate tumor and stromal cells, and demonstrated that the two compartments are characterized by distinct gene expressions, both in the native state and following HBO treatments. Furthermore, hyperoxia induced a significant tumor growth-inhibitory effect, with significant down-regulation of the MAPK pathway. An anti-angiogenic effect after intermittent HBO was observed, and reflected in the gene expression profile.

## Background

The tumor microenvironment is increasingly recognized as a pivotal factor in tumor progression [1], and studies show that the tumor stroma strongly influences angiogenesis and vascular permeability [2-4]. Understanding the biological heterogeneity in primary cancers and their metastases, and the process by which tumor cells invade distant tissues, is necessary to develop effective cancer therapies [5]. The non-obese diabetic/severe combined immunodeficient (NOD/SCID) mice expressing enhanced-green fluorescent protein (eGFP), combined with dsRed transfected tumor cells enables studies of tumor-stroma cell interactions, both *in situ *and *ex vivo *[6]. Fluorescence-activated cell sorting (FACS) enables complete separation of green stromal cells from red tumor cells and provides a system for detailed analysis of tumor-stroma interactions.

Hypoxia activates signalling pathways that regulate cellular proliferation, angiogenesis and cell death [7]. Adaptation to these pathways allows cancer cells to survive and even grow under hypoxic conditions. The fact that tumors contain hypoxic areas was discovered nearly sixty years ago and was shown to correlate with poor response to radiotherapy [8,9]. Later, hypoxia has also been shown to decrease the efficacy of chemotherapy and has been associated with a poor treatment outcome [10,11].

Due to the tumor-promoting effects of hypoxia, a reduction in the hypoxic state of the tumor might have an inhibitory effect on tumor growth. Previously, induction of hyperoxia by hyperbaric oxygen (HBO), have demonstrated successful growth inhibition and potentiation of the chemotherapeutic effect [12-16]. HBO is based on 100% oxygen exposure at a pressure level higher than normal atmospheric pressure, thereby enhancing the amount of dissolved oxygen in the plasma [17].

We aimed to establish a model system for studying tumor-stroma interactions in 4T1 mammary tumors. This model enables separation of eGFP labelled stromal cells from dsRed transfected 4T1 mammary tumor cells, and provides an opportunity to elucidate changes in gene expression in the two compartments. Furthermore, using this model we aimed to study the biological effects of enhanced oxygenation on tumor growth and regression.

## Methods

### Cell line and culture conditions

The murine mammary cell line 4T1 (American Type Culture Collection, Rockville, MD, USA) was transfected with red fluorescent protein using a dsRed-expressing lentiviral vector. This cell line was originally isolated from a spontaneously arising mammary tumor in BALB/cfC3H mice [18]. Successful transfection with dsRed was confirmed by fluorescence microscopy (Axiolmager 2, Carl Zeiss MicroImaging, GmbH, Jena, Germany). 4T1 cells were cultured in RPMI-1640 medium (Bio-Whittaker, Verviers, Belgium) supplemented with 10% Foetal Calf Serum (Sigma-Aldrich, Steinheim, Germany), 100 units/ml penicillin, 100 μg/ml streptomycin, 2% L-glutamine (All from Bio-Whittaker, Verviers, Belgium). The cells were maintained at 37°C in 5% CO_2 _and 95% air, and were seeded and used at ~ 80% confluence in all experiments.

### Ethics Statement

All the animal experiments were performed in accordance with the regulations of the Norwegian State Committee for Animal Research ("Forsøkdyrutvalget", approval number 1279) and approved by the Local Institution Board at the University of Bergen (approval number 2008076BB). The investigation conforms to the Guide for the Care and Use of Laboratory Animals published by the US National Institutes of Health.

### *In vivo *experiments

Female NOD/SCID mice (18-24 g) were used in this study. Generally, the NOD/SCID mice used expressed eGFP in all nucleated cells, the exception being histological and immunohistochemical experiments, where plain NOD/SCID mice were used. The emission of green fluorescence has previously been observed in muscle, pancreas, kidney, heart and other organs of the mice, confirming the fluorescent phenotype [6]. The breeding was performed at the animal facility at the University of Bergen, as described by Niclou *et al. *[6]. All animal experiments were performed under Isoflurane (Rhone-Puolenc Chemicals, France) and N_2_O gas-anaesthesia. A 17β-estradiol pellet (0.18 mg/pellet--60 day release, Innovative Research of America, Sarasota, FL, USA) was inserted into the interscapular area of all mice prior to tumor cell injection. The pellets provide a continuous release of estradiol to give serum concentrations of 150-250 pM. 3 × 10^6 ^4T1 cells dissolved in 0.15 ml PBS were injected in the mouse mammary fat pad, in the groin area. 4T1 tumors had a 100% take rate, and a 6 days latency period to a palpable tumor estimated to be ~5 mm in diameter.

The tumors were measured externally with a calliper at day 1 (*pre *HBO exposure), day 4 and 8 (*post *HBO exposure). The location of the tumor excluded external measurement in more than two dimensions. The tumor volume was therefore calculated assuming a cylindrical shape of the tumor, according to the formula: π/6 ·a^2^·b, where a is the shortest and b is the longest transversal diameter.

### HBO treatment

A 27 l Hyperbaric Animal Research Chamber (OXYCOM 250 ARC, HYPCOMOY, Tampere, Finland) with an inner diameter of 25 cm, and an inner length of 55 cm was used. The chamber was supplied with pure O_2_. After reaching 100% O_2 _(15 min), the pressure was raised over a period of approximately 5 min to 2.5 bar (equivalent to 15 meters sea water). The 2.5 bar pure oxygen atmosphere was maintained for a period of 90 min. To maintain > 97% O_2 _atmosphere, the chamber was flushed with pure oxygen for 3-5 min every 10-30 min depending on the number of mice in the chamber. After treatment, the mice were slowly decompressed over a 10 min period.

Three separate groups of mice were studied. The first group of tumor bearing mice was exposed to intermittent HBO treatment (2.5 bar and 100% O_2_, 3 exposures à 90 min on day 1, 4 and 7). The second group was exposed to daily HBO treatments (2.5 bar and 100% O_2_, 7 daily exposures à 90 min), whereas the control group was housed under normal atmosphere for the experimental period of 8 days.

### *In situ *and *ex vivo *imaging

We used a fluorescence dissection microscope (Model C-DSD230, Nikon, Japan) with UV-filter optics for dsRed and eGFP, to observe the tumor *in situ*. After the mice were sacrificed with saturated KCl during anaesthesia, the tumors were excised. The tumors were processed in three different ways: 1) formalin (4%), and later embedded in paraffin. 2) frozen in liquid nitrogen and stored at -80°C until further use. 3) paraformaldehyde (PFA) prior to freezing, and then embedded in Prolong Gold (Invitrogen, CA, USA) after sectioning. PFA fix was performed to conserve the fluorescent traits, when visualized under the microscope (Leica TCS SP5, Wetzlar, Germany).

### Histology and immunohistochemistry

Paraffin embedded tumor sections from all three groups were H&E stained, and examined by an experienced pathologist.

Frozen tumor sections (20 μm) were used for immunostaining of blood vessels using rat anti-mouse CD31 (AbD Serotec, Morphosys UK Ltd, Oxford, UK) as primary antibody and biotinylated rabbit anti-rat as secondary antibody (Vectastatin ABC kit, peroxidase IgG PK 4004, Bioteam AS, Trondheim, Norway). An ABC kit (Vectastatin ABC kit, peroxidase IgG PK 4004, Bioteam AS, Trondheim, Norway) and Diaminobenzidine tetrahydrochloride (3.3 DAB, Sigma Aldrich, Germany) were used as a chromogen to visualize blood vessels. Richardson stain was used as a nuclear counterstain. The cross-sectional density of CD31 positive structures was quantified per mm^2 ^using a counter grid, covering the viable tumor area. Tumor cell proliferation was assessed by staining with an anti-rabbit Ki67 antibody diluted 1:100 (Millipore, Billerica, MA), and biotinylated goat anti-rabbit secondary antibody (DACO Patts, Glostrup, Denmark) on frozen tumor sections. Four Ki67 labelled "hot spots" within selected high power fields of view (HPFs) were quantified using the NIS-Elements^™ ^BR 3.1 software (Nikon Corporation, Tokyo, Japan), under 400× magnification. The immunopositive cells were counted and expressed as a fraction (%) of the total cells. Four fields of vision per section from each animal were included in the quantification. Cell death was examined by the terminal transferase-mediated dUTP nick-end-labeling (TUNEL) method (Boehringer Mannheim, Mannheim, Germany), performed according to the manufacturers recommendation on frozen tumor sections. For quantifying TUNEL labelled cells, threshold levels of pixel intensity were determined and expressed as % positive cells/area fraction at x200 magnification. All sections were examined using a Nikon light microscope (THP Eclipse E600, Nikon Corporation) and the images were captured with a Nikon Digital Camera (DXM 1,200F, Nikon Corporation). The quantification was performed blindly.

### Fluorescence-activated cell sorting (FACS)

Freshly isolated 4T1 tumors were dissociated by mincing the tissue with scalpels, followed by incubation with 1 mg/ml collagenase/dispatase (Roche Diagnostics GmbH, Germany) and 0.125% DNase I (Sigma-Aldrich) dissolved in DMEM medium for 60-90 min at 37°C. Incompletely dissociated tissue was digested a second time using the same procedure. The dissociated tumor tissue was then washed with ice-cold FACS buffer (PBS with 2% FBS) and filtered twice through a 70-μm cell strainer. The cell suspension was then centrifuged at a speed of 500 G for 10 min (4°C). The cell pellets were resuspended in FACS buffer for further analysis and sorting. The single cell suspension was filtered through a 40-μm cell strainer in order to remove any clumping cells before sorting. The cells were sorted using a cell sorter (FACS Aria SORP, BD Biosciences, Erembodegem, Belgium) based on the single-cell viability and the fluorescence intensity of eGFP and dsRed, and separation was confirmed by fluorescence microscopy (Nikon ellipse 2000, Nikon, Japan).

### RNA isolation and quantification

Sorted cells were lysed using 350 μl Buffer RLT Plus with β-mercaptoethanol (β-ME). The lysate was homogenized by passing it 5 times through a 23 G needle. Further, the protocol for purification of total RNA from animal cells (RNeasy Plus Mini Handbook, Qiagen AB, Sweden) was used according to the manufacturer's recommendations. RNA concentration (ng/μl) and purity (260/280 ratio) were determined using NanoDrop 1,000 Spectrophotometer (Thermo Scientific, Sweden).

### Gene expression analysis

Global gene expression analysis was performed in order to identify key molecular mechanisms and differences in gene programs in stromal and tumor cells, as well as changes following HBO treatment. A total of 18 tumor bearing mice were used. Seven mice served as control animals (non-HBO exposure), 5 mice were HBO treated intermittently and 6 mice treated daily. Stromal cells and tumor cells from the different tumors were isolated as described above. Total RNA purification, cRNA labeling, microarray hybridization and features extraction were performed as previously described [14]. The Agilent G4122F Whole Mouse Genome (4 × 44 k) Oligo Microarray Kit with SurePrint Technology (Agilent Technologies, Inc., Palo Alto, CA) was used to analyze samples in the present study. The normalized channel values were log (2) transformed and combined into a gene expression data matrix. Data were formatted in a J-Express-file suitable for additional data mining (http://www.molmine.com/) [19]. Following normalization, the treated tumor cells and stromal cells were analysed against the respective non-treated controls of stromal and tumor cells. We also analysed untreated stromal cells against untreated tumor cells to highlight and characterize the stromal cell signatures. A similar approach was performed to analyse how the treated tumor cells responded to HBO treatment versus treated stromal cells (results not shown).

### Statistical analysis

For the gene expression data, we used analysis of variance (ANOVA), SAM (Significant Analysis of Microarray), GSEA (Gene Sets Enrichment Analysis) [20] and Gaussian kernels of the J-Express program package for identification of differentially expressed genes. Following SAM we selected genes with False Discovery Rate (FDR) < 5% as basis for GSEA and Hierarchical clustering. Gene sets consisting of more than 18 genes and less than 200 genes were selected for further analysis of cellular processes, pathways and molecular function. Annotated microarray data were uploaded in the BASE database and formatted and exported to ArrayExpress at the European Bioinformatics Institute (http://www.ebi.ac.uk/arrayexpress (Accession number: E-TABM-1103)) in agreement with the MIAME guidelines. Two-tailed unpaired t-test (normalized data) or the non-parametric Mann-Whitney test (non-normalized data) was used for testing the statistical differences between groups. *P *< 0.05 was considered statistically significant. GraphPad InStat 3 (GraphPad Software, Inc., La Jolla, USA) and SPSS for Windows (IBM Corporation, NY, USA) were used for the statistical analysis.

## Results

### Fluorescent imaging

**Figure 1:**
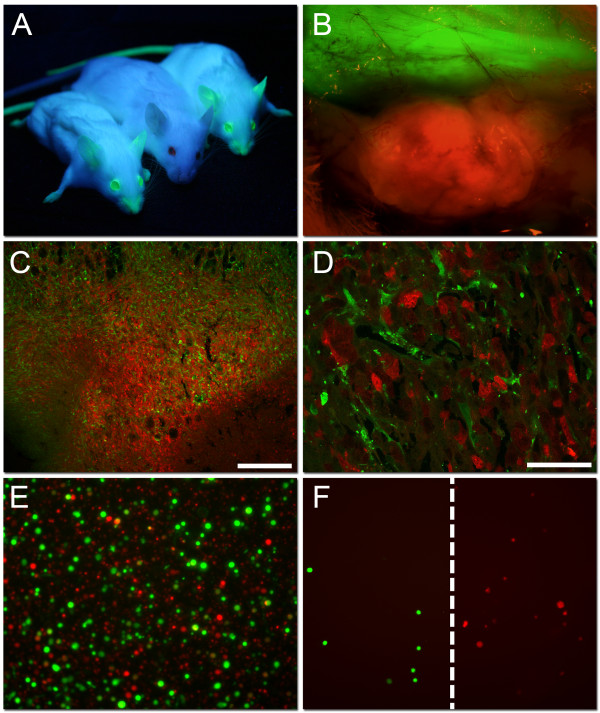
**dsRed transfected 4T1 mammary tumor in eGFP mice**. A) Two NOD/SCID mice expressing enhanced green fluorescent protein (eGFP) under UV-illumination flanking one plain NOD/SCID mouse from the non-transgenic parental line. B) An *in situ *picture of a 4T1 dsRed tumor growing subcutaneously in the NOD/SCID eGFP expressing mouse after removing the skin flap (×40 magnification). C and D) Representative confocal microscopy pictures of a 4T1 mammary control tumor, with dsRed expressing tumor cells, as well as infiltrating eGFP expressing host cells. Scale bars indicate 250 μm (C) and 50 μm (D). E) The dissociated 4T1 mammary tumor showing single eGFP expressing host cells together with single dsRed transfected tumor cells (×100 magnification). F) eGFP expressing stromal cells and dsRed expressing 4T1 tumor cells after Fluorescence activated cell sorting (FACS), verify successful separation.

To verify tumor growth in the eGFP mice (Figure 1A), a fluorescence dissection microscope with UV-filter optics for dsRed and eGFP was used. As shown in Figure 1B, a red 4T1 mammary tumor was seen within the surrounding green host tissue. The overall tumor surface architecture was visualized, and blood vessels within the tumor bed appeared darker. The vasculature in the skin flap as well as the transition areas between tumor tissue and host tissue was also clearly visualized. Thus, dsRed transfected 4T1 mammary tumors were successfully established subcutaneously (sc) in the eGFP mice. Confocal microscopy revealed that the tumors contained both red tumor cells as well as infiltrating eGFP expressing stromal cells (Figure 1C and 1D). Before separating the tumor cells from the stromal cells using FACS, the tumor bulk was dissociated (Figure 1E). Further, a successful separation of the green stromal cells from the red tumor cells was confirmed by fluorescence microscopy (Figure 1F).

### Tumor growth

**Figure 2:**
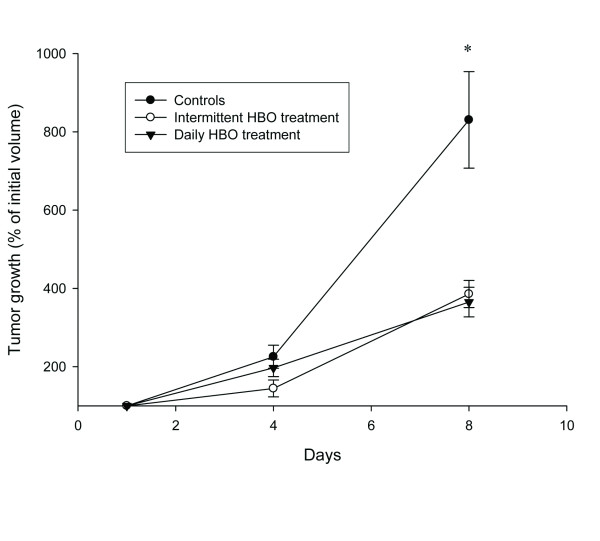
**Tumor growth**. 4T1 mammary tumor growth (% of initial volume) in control (n = 19), intermittent (n = 24) and daily (n = 9) hyperbaric oxygen (HBO) treated tumors during an 8 day period. Intermittent treatments were given at day 1, 4 and 7. Data represent mean ± SEM. * *p *< 0.001 compared to control.

Both untreated and HBO treated 4T1 mammary tumors were measured. Six days after injection of the 4T1 cells, the average size of all tumors were approximately 80 mm^3 ^(day 1). The control tumors (n = 19) increased in size with ~800% within the first 8 days of tumor development (Figure 2). Exposing the mice to 2.5 bar pure oxygen (n = 24), on days 1, 4 and 7, each for 90 min, inhibited tumor growth significantly compared to untreated control tumors during the same time period (*p *< 0.001) (Figure 2). A similar reduction was found after daily HBO exposure (n = 9) 7 times (*p *< 0.001). Thus, enhanced oxygenation significantly inhibited the growth of the 4T1 mammary tumors.

### Tumor morphology

Control 4T1 tumors (n = 7) were highly cellular and revealed an undifferentiated morphology showing both epitheloid and spindle shaped tumor cells, marked nuclear atypia and numerous mitotic figures, with diffuse growth in the surrounding fat tissue. They also showed minimal to extensive (~ 70-80%) necrosis with areas of granulation tissue. In the tumors treated with intermittent HBO (n = 5) and daily HBO (n = 9), no clear differences from control tumors were evident.

### Tumor blood vessels

Angiogenesis is pivotal for tumor growth, and may be a strong contributor to the differences in tumor size observed between controls and the HBO treated groups. We therefore quantified the average number of CD31 positive tumor blood vessels (blood vessels/mm^2^). A concomitant study from our group demonstrated that intermittent HBO significantly decreased tumor blood vessel density compared to controls (~50%), (Table 1) [21]. However, we now demonstrate that the tumor blood vessel density is not affected by 7 daily treatments of HBO. Thus, only intermittent HBO (3 HBO exposures) has a strong anti-angiogenic effect on the 4T1 mammary tumors.

**Table 1:** Immunohistochemical analysis

	Control	Intermittent HBO	Daily HBO
**Angiogenesis **(numbers/mm^2^)	79.9 ± 18.7^Δ^	41.8 ± 17.1*^Δ^	79.2 ± 16.2
**Proliferation** (% Ki67-positive cells)	21.7 ± 17.6	26.5 ± 17.9	21.4 ± 7.8
**Cell death** (% of total area)	7.6 ± 4.1	7.8 ± 1.3	5.8 ± 0.8

Angiogenesis (CD31), proliferation (Ki67) and cell death (TUNEL) stained in tumors from untreated controls and after intermittent and daily hyperbaric oxygen treatment (HBO). n = 5 in all groups. Mean values ± SD. *p < 0.02 compared to both control and daily HBO treatment. ^Δ^results obtained from Jevne *et al. *[21]

### Cell death and proliferation

The balance between cell proliferation and cell death is an important factor deciding the tumor growth rate. We therefore aimed to investigate the amount of cell death by using TUNEL staining and proliferation by using Ki67 staining. The results showed that there were no significant differences between the three experimental groups, neither in proliferation nor cell death, when counted in hot spot areas (Table 1).

### Gene expression analysis

**Figure 3:**
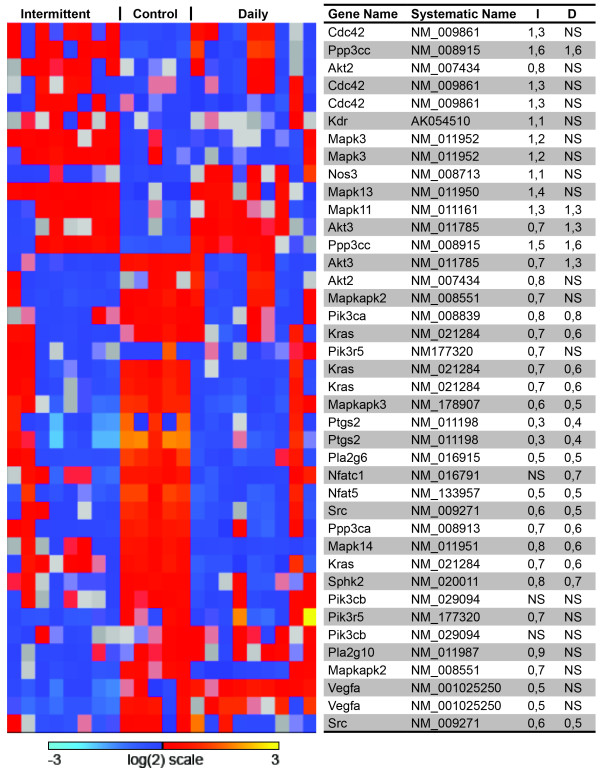
**VEGF pathway, tumor**. Heatmap of genes in the VEGF signaling pathway (HSA04370). The table display gene names, systematic names, and fold change of gene expression compared to control of intermittent (I) and daily (D) hyperbaric oxygen (HBO), respectively. The VEGF pathway is significantly down-regulated by intermittent HBO treatment of 4T1 mammary tumor cells, but not by daily treatment. NS = not significant.

**Figure 4:**
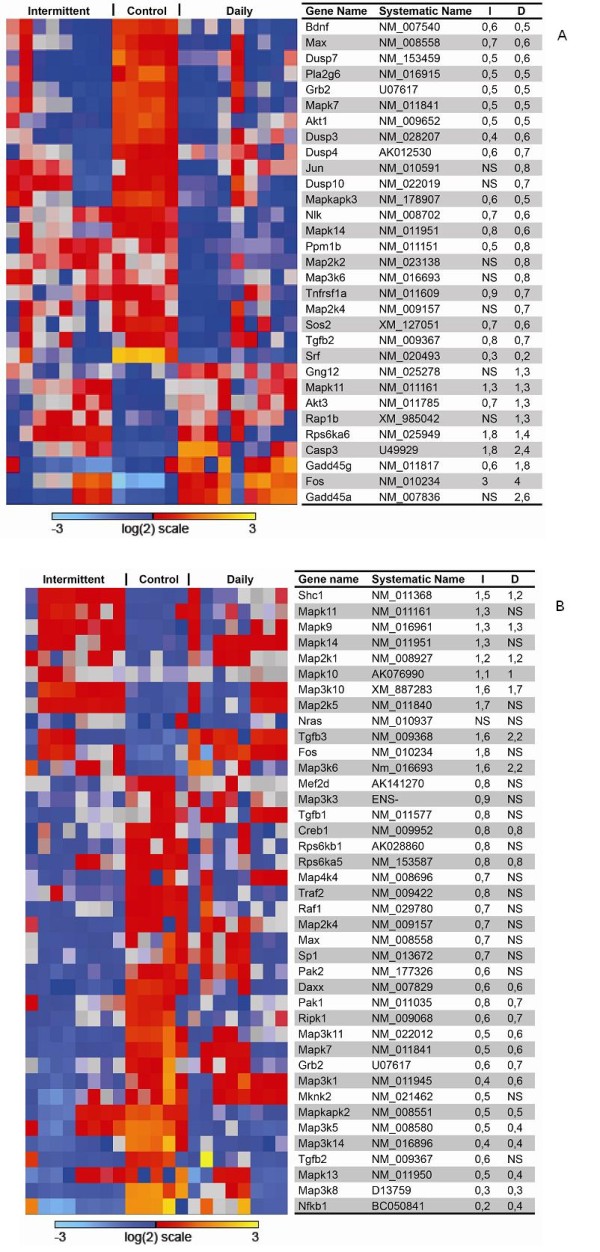
**MAPK pathway, tumor (a) and stroma (b)**. Heatmap of genes in the MAPK pathway (HSA04010). The table display gene names, systematic names, and fold change of gene expression compared to control of intermittent (I) and daily (D) hyperbaric oxygen (HBO), respectively. Intermittent and daily HBO treatment suppressed MAPK signaling pathway in both tumor cells and stromal cells compared to control. NS = not significant.

The purity of sorted cells was verified by fluorescence microscopy. Additionally, gene expression profiling demonstrated clear separation based on the expression of mesenchymal markers in the stromal cells (Table 2). Additional file 1: Figure S1 displays the number of genes significantly changed after treatment compared to controls. 5,195 genes in the tumor cell compartment were changed both following HBO daily and intermittent treatment. In the stromal cell compartment, 4,802 genes were changed following both treatments. Additional file 2: Table S1, Additional file 3: Table S2, Additional file 4: Table S3, Additional file 5: Table S4 summarize the results from a Gene Set Enrichment Analysis (GSEA), clustering the important signalling pathways significantly changed in tumor cells and stromal cells after intermittent and daily HBO treatment, respectively. The expression of angiogenic markers in both tumor and stromal cells after intermittent and daily HBO were compared to untreated tumor and stromal cells (Table 3), demonstrating that in this study, enhanced oxygenation induced a down-regulation of pro-angiogenic genes both in tumor and stromal cells in the intermittent group. Moreover, in the daily HBO treated group, expression of pro-angiogenic genes was down-regulated in tumor cells, although fewer genes were significantly changed. Figure 3 displays a heat map of the VEGF-signalling pathway (HSA04370), showing that this important pro-angiogenic pathway is down-regulated in tumor cells after intermittent HBO, but not significantly changed in tumor cells after daily HBO. The MAPK pathway (HSA04010) was significantly down-regulated in both tumor and stromal cells after daily and intermittent HBO compared to untreated control (Figure 4a and 4b).

**Table 2:** Gene-signature of stromal cells

Epithelial markers in stroma cells		Mesenchymal markers in stromal cells	
***Krt14***	0.3	***Snai1***	2
***Cdh1***	0.2	***Snai2***	19
***Perp***	0.3	***Fgfr1***	7
***Dsp***	0.1	***Cdh2***	2.3
***Ocln***	0.2	***Cdh11***	16
***Cdh3***	1.3	***Twist2***	25
		***Nid1***	115

The numerals are referred to as fold change in gene expression of untreated stromal cells as compared to untreated tumor cells. All values: FDR < 0.00%

**Table 3:** Markers of angiogenesis

		Intermittent HBO		Daily HBO	
		Tumor	Stroma	Tumor	Stroma
Genes	Gene name	Fold change	Fold change	Fold change	Fold change
**Anti-Angiogenic**					

***Serpinb5***	Serine (or cysteine) peptidase inhibitor, clade B, member 5	**3.4**	**1.6**	**5.0**	NS
***Csta***	Cystatin A	**0.1**	NS	NS	NS
***Thbs1***	Thrombospondin1	**0.5**	**0.5**	NS	NS
***Thbs2***	Thrombospondin2	NS	**2.7**	NS	**2.5**
***Thbs3***	Thrombospondin3	NS	NS	**0.7**	NS
***Thbs4***	Thrombospondin4	**0.8**	NS	NS	NS

**Pro-angiogenic**					

***HIF1a***	Hypoxia-inducible factor 1, alpha	NS	NS	**0.7**	NS
***Pecam1***	Platlet/endothelial cell adhesion molecule 1	**0.9**	**0.5**	**0.6**	NS
***Vwf***	Von Willebrand factor homolog	**0.7**	**0.3**	NS	**0.6**
***Vegfa***	Vascular endothelial growth factor A	**0.5**	**0.5**	NS	**1.4**
***Vegfb***	Vascular endothelial growth factor B	NS	NS	**0.7**	NS
***Vegfc***	Vascular endothelial growth factor C	**0.6**	NS	**0.5**	NS
***Pdgfa***	Platelet derived growth factor, alpha	**0.8**	**0.7**	NS	NS
***Pdgfb***	Platelet derived growth factor, b	**1.2**	**0.7**	NS	NS
***Pdgfra***	Platelet derived growth factor receptor, alpha	**0.7**	**2.3**	**0.5**	**2.1**
***Tgfb1***	Transforming growth factor, beta 1	NS	**0.8**	**0.8**	NS
***Il6***	Interleukin 6	**0.5**	NS	NS	**0.3**
***Fgfr1***	Fibroblast growth factor receptor 1	**0.9**	NS	NS	NS
***Fgfr3***	Fibroblast growth factor receptor 3	**0.7**	NS	**0.7**	NS
***Egfr***	Epidermal growth factor receptor	**0.5**	NS	**0.5**	**2.7**
***Serpinb2***	Serine (or cysteine) peptidase inhibitor, clade B, member 2	**0.6**	NS	NS	**0.5**

Displayed results are fold change of gene expression in intermittent and daily hyperbaric oxygen (HBO) treated tumors cells compared to untreated control tumor cells, and intermittent and daily HBO treated stromal cells compared to untreated stromal cells. A positive fold change indicates increased expression after treatment. FDR < 0.05%, NS = not significant

## Discussion

### Tumor and stroma in untreated control tumors

Proliferation, survival and migration of breast cancer cells can be modulated by stromal cells [22]. Microarray studies have made it possible to molecularly classify breast cancers, and correlate their signatures with metastatic behaviour and clinical outcome [23]. Nevertheless, since most of the *in vivo *studies have been performed on tumor tissue homogenates that provides material based on all cell types it does not allow for separate assessment of the tumor cells and stromal compartments in solid tumors, and so, subtle changes within one compartment may be masked by the bulk of surrounding cells. Thus, by using red 4T1 cells injected in green mice, we were able to investigate complex tumor-stroma interactions, making a novel contribution to the studies of tumor-host interactions in mammary tumors.

We have developed a mammary tumor model in immunodeficient mice in order to separate the stromal cells from the tumor cells, as indicated in Figure 1B. Fluorescence microscopy (Figure 1F) showed a successful separation of the green stromal cells from the red tumor cells by FACS. Stromal cells expressed mesenchymal markers, as indicated in Table 2, confirming their origin. Gene expression results in the untreated control tumors also verify expression-patterns of stromal cells, with high expression of ECM genes, such as collagens and integrins, in addition to high expression of MMP's and chemokines involved in invasion and metastasis (data not shown).

### Changes in tumor and stroma after enhanced oxygenation

The present study aimed to elucidate the effect of enhanced oxygenation on tumor growth and tumor-stroma interactions. Previous studies from our laboratory have shown a significant tumor inhibitory effect of intermittent HBO on DMBA (dimetyl-α-benzantracene)-induced mammary tumors [12,14,16]. Thus, we wanted to expand our study of enhanced oxygenation to another mammary tumor model, to determine if HBO has a general inhibitory effect on breast cancer. Both treatment regimens had a significant growth-inhibitory effect on the present 4T1 tumor model. However, the inhibitory effect was not as pronounced in the present 4T1 tumors as for the DMBA-induced tumors [12,14,16].

The number of genes significantly changed after treatment compared to control is shown in Additional file 1: Figure S1. To be able to systematize the gene expression results, we have searched for important signalling pathways by GSEA. Signalling pathways significantly changed in tumor cells and stromal cells after intermittent and daily HBO treatment, respectively, is shown in Additional file 2: Table S1, Additional file 3: Table S2, Additional file 4: Table S3, Additional file 5: Table S4. The results will be discussed further in the following sections.

#### Angiogenesis

The importance of angiogenesis in tumor growth and progression is well known, so a significant reduction in tumor blood vessel density might be an important factor explaining the tumor inhibitory effect after HBO. Immunohistochemistry demonstrates a significant anti-angiogenic effect after intermittent HBO treatment (Table 1). This corresponds to the anti-angiogenic effect shown previously on both DMBA-induced mammary tumors and gliomas after intermittent HBO treatment [13,14]. Unexpectedly, immunohistochemistry did not show the same anti-angiogenic effect after daily HBO. Table 3 displays important pro- and anti-angiogenic factors, and show changes in gene expression in both tumor and stromal cells treated with either intermittent or daily HBO compared to untreated tumor or stromal cells. The gene expression data indicate a gene regulatory trend supporting the results from immunohistochemistry. In general, pro-angiogenic genes are down-regulated after both intermittent and daily HBO. However, fewer genes are significantly changed in the daily HBO treated group. Figure 3 displays a heatmap of the VEGF-signalling pathway (HSA04370), showing that this important pro-angiogenic pathway is down-regulated in tumor cells after intermittent HBO, but not significantly changed after daily HBO. However, the stromal compartment does not show the same trend. As we observe an anti-angiogenic effect in the intermittent group, we might speculate that the stromal influence is not the most important contributor to angiogenesis, as the tumor cell compartment seems to be more influenced by hyperoxia.

As both HBO groups display tumor growth retardation, whilst only the intermittent HBO treated group influences angiogenesis, we decided to expand the treatment-time of the two groups, in two separate series, to determine if tumor growth and angiogenesis were changed after long-term treatments (Additional file 6: Figure S2). In the extended experiment, one group of animals was treated with 5 intermittent HBO treatments (Day 1, 4, 7, 10 and 13), whilst the daily group was treated every day for 13 days. Tumor growth measurements and CD31 staining of the two long-term HBO treated groups displayed no significant differences between the two groups, indicating that other factors must be involved.

#### Proliferation and apoptosis

Immunohistochemical analysis of proliferation and cell death showed no significant changes in neither intermittent nor daily HBO when compared to the untreated control tumors in hot spot areas (Table 1). However, GSEA showed changes in important cellular pathways (Additional file 2: Table S1, Additional file 3: TableS2, Additional file 4: Table S3, Additional file 5: Table S4). MAPK (HSA04010) is down-regulated in both cellular compartments after HBO treatments, although not markedly changed (Figure 4a and 4b). MAPK has been shown to be important for proliferation, differentiation, migration and apoptosis [24]. *Grb2 *is significantly down-regulated in both tumor and stroma after both HBO treatments. As GRB2 is an adaptor protein involved in signal transduction and cell communication, down-regulation of this gene will impede MAPK-signal transduction. This might indicate changes in the tumor after HBO that we were not able to verify with our immunohistochemical analysis, and that these changes might support the growth-inhibitory effect.

#### Invasion and metastasis

Tao *et al. *[25] studied tumor growth and metastasis in the 4T1 mammary tumor model using biophotonic imaging. 4T1 cells metastasise to sites affected in human breast cancer, like lungs, liver, brain and bone [25]. In the present study, all mice in the supplementary long-term HBO treatments groups (5 × HBO or 13 × HBO), 20 days after injection, had lung metastases (Additional file 6: Figure S2). This corresponds to the findings of Tao *et al. *[25] where lung metastases were found 15-21 days after injection. However, since we do not have untreated control tumors at this time point, we can only speculate on whether or not HBO hinder metastasis. Haroon *et al. *[26] found a significant decrease in large colony lung metastases after HBO treatment, indicating that HBO restricts the growth of large tumor cell colonies.

## Conclusion

The present *in vivo *4T1 mammary tumor model enabled us to completely separate tumor cells from stromal cells. The data demonstrated that the two compartments are characterized by distinct differences in gene expressions both in the native state and following hyperoxic treatment. Furthermore, hyperoxia induced a significant tumor growth-inhibitory effect, with a significant down-regulation of the MAPK pathway. After intermittent hyperoxic treatment, an anti-angiogenic effect was observed and reflected in expression trends of angiogenic genes.

## Abbreviations

ANOVA: Analysis of variance; DMBA: Dimetyl-α-benzantracene; ECM: Extracellular matrix; eGFP: Enhanced-green fluorescent protein; FACS: Fluorescence-activated cell sorting; FDR: False discovery rate; GSEA: Gene sets enrichment analysis; HBO: Hyperbaric oxygen; H&E: Hematoxylin & eosin; NOD/SCID: Non-obese diabetic/Severe combined immunodeficient; PFA: Paraformaldehyde; SAM: Significant analysis of microarray; sc: Subcutaneously; TUNEL: Terminal transferase-mediated dUTP nick-end-labeling. The abbreviated gene names are fully described in the tables.

## Competing interests

The authors declare that they have no competing interests.

## Authors' contributions

IM and CJ carried out the cell and animal handling, implantation, HBO treatments, tumor growth measurements, CD31 immunohistochemistry and the preparation for FACS. Additionally, IM analysed and interpreted the data and drafted the manuscript. JW performed the FACS experiments. LS purified the RNA. MC stained and quantified ki67 and TUNEL sections. LAA examined the histological sections. AMØ and KHK performed and analysed the microarray data. PØE, RKR and LEBS participated in interpretation of data and manuscript drafting. Additionally, LEBS conceived the idea and was in charge of the study design. All authors read, commented on and approved the final manuscript.

## Pre-publication history

The pre-publication history for this paper can be accessed here:

http://www.biomedcentral.com/1471-2407/12/21/prepub

## Supplementary Material

Additional file 1 **Figure S1 Significantly changed genes in the tumor and stromal compartment**. The number of significantly expressed genes (FDR < 5%) in tumor cells and stromal cells treated with daily and intermittent hyperbaric oxygen (HBO), both compared to control cells. Overlapping genes in the two different compartments are shown in red. Click here for file

Additional file 2 **Table S1**. Cellular processes, pathways and molecular function. Gene set enrichment analysis (GSEA) after intermittent hyperbaric oxygen (HBO) treatment of tumor cells. Click here for file

Additional file 3 **Table S2**. Cellular processes, pathways and molecular function. Gene set enrichment analysis (GSEA) after daily hyperbaric oxygen (HBO) treatment of tumor cells. Click here for file

Additional file 4 **Table S3**. Cellular processes, pathways and molecular function. Gene set enrichment analysis (GSEA) after intermittent hyperbaric oxygen (HBO) treatment of stroma cells. Click here for file

Additional file 5 **Table S4**. Cellular processes, pathways and molecular function. Gene set enrichment analysis (GSEA) after daily hyperbaric oxygen (HBO) treatment of stroma cells. Click here for file

Additional file 6 **Figure S2 Tumor growth and metastasis in long-term HBO treated tumors**. 4T1 mammary tumor growth (% of initial volume) over 14 days in long-term intermittent (n = 7) and long-term daily (n = 7) hyperbaric oxygen (HBO) treated tumors. Intermittent treatments were given at day 1, 4 and 7, 10 and 13. Data represent mean ± SEM. B) Histological section displaying lung metastasis after (B) long-term intermittent HBO and (C) long-term daily HBO treatment (both × 100). Click here for file
