# Supplementary figures and images for: Gene expression in tumor cells and stroma in dsRed 4T1 tumors in eGFP-expressing mice with and without enhanced oxygenation
Source: BMC Cancer. 2012 Jan 17;12:21. doi: 10.1186/1471-2407-12-21 (PMC3274430; doi:10.1186/1471-2407-12-21)

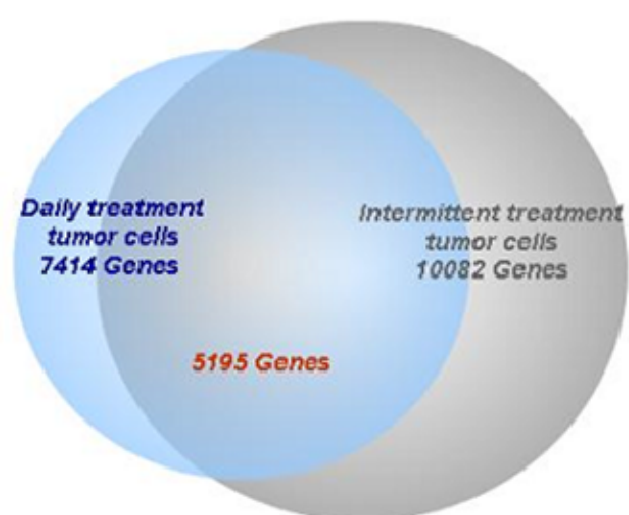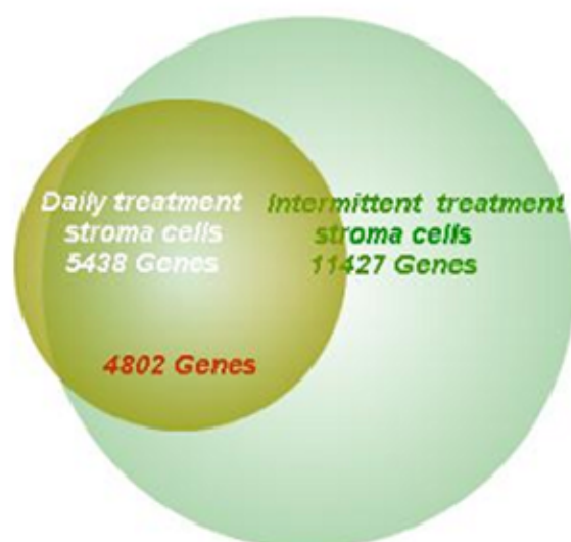

Supplement: Additional file 1 — Figure S1 Significantly changed genes in the tumor and stromal compartment. The number of significantly expressed genes (FDR < 5%) in tumor cells and stromal cells treated with daily and intermittent hyperbaric oxygen (HBO), both compared to control cells. Overlapping genes in the two different compartments are shown in red. [file 1471-2407-12-21-S1.PDF]

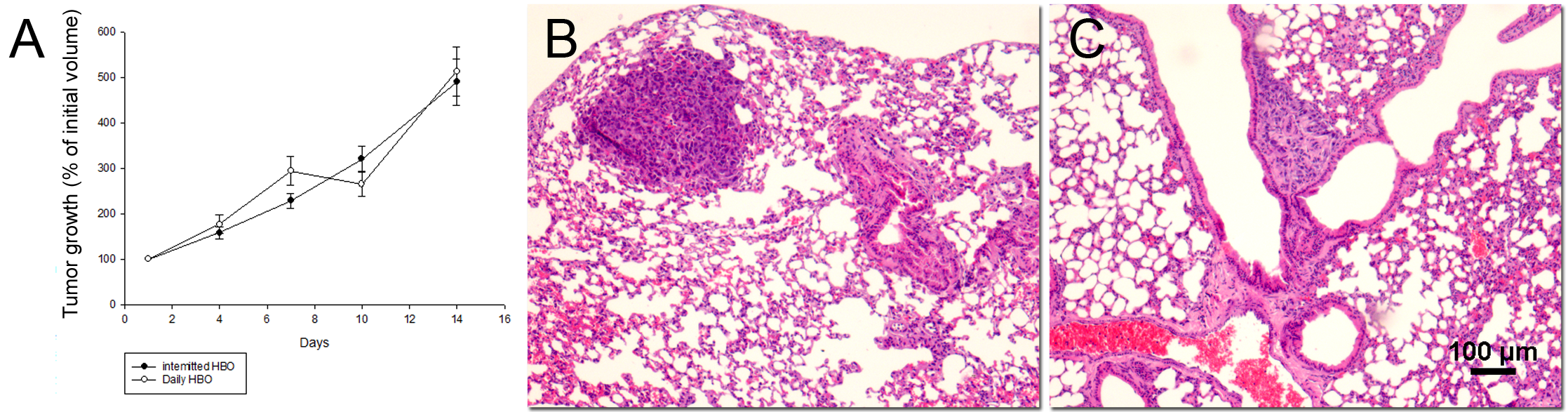

Supplement: Additional file 6 — Figure S2 Tumor growth and metastasis in long-term HBO treated tumors. 4T1 mammary tumor growth (% of initial volume) over 14 days in long-term intermittent (n = 7) and long-term daily (n = 7) hyperbaric oxygen (HBO) treated tumors. Intermittent treatments were given at day 1, 4 and 7, 10 and 13. Data represent mean ± SEM. B) Histological section displaying lung metastasis after (B) long-term intermittent HBO and (C) long-term daily HBO treatment (both × 100). [file 1471-2407-12-21-S6.TIFF]
