# Supplementary material for: Gene expression in tumor cells and stroma in dsRed 4T1 tumors in eGFP-expressing mice with and without enhanced oxygenation
Source: BMC Cancer. 2012 Jan 17;12:21. doi: 10.1186/1471-2407-12-21 (PMC3274430; doi:10.1186/1471-2407-12-21)
Supplement: Additional file 2 — Table S1. Cellular processes, pathways and molecular function. Gene set enrichment analysis (GSEA) after intermittent hyperbaric oxygen (HBO) treatment of tumor cells. [file 1471-2407-12-21-S2.PDF]

**Table S1:** Cellular processes, pathways and molecular function. Gene set enrichment analysis (GSEA) after intermittent hyperbaric oxygen (HBO) treatment of tumor cells.

| **Induced_intermittent HBO treatment** | **FDR** | **No.genes** |
|---|---|---|
| HSA00190_OXIDATIVE_PHOSPHORYLATION | 0.00 | 51 |
| ELECTRON_TRANSPORT_CHAIN | 0.15 | 53 |
| PROTEIN_SERINE_THREONINE_KINASE_ACTIVITY_DN | 0.85 | 100 |
| ACTIN_CYTOSKELETON | 1.80 | 62 |
| G1_TO_S_CELL_CYCLE_REACTOME | 1.96 | 30 |
| HDACI_COLON_BUT16HRS_DN | 2.23 | 45 |
| METALLOENDOPEPTIDASE_ACTIVITY | 2.30 | 18 |
| NEGATIVE_REGULATION_OF_CELL_CYCLE | 2.52 | 30 |
| HSA03320_PPAR_SIGNALING_PATHWAY | 2.83 | 20 |
| **Down_intermittent HBO treatment** | **FDR** | **No.genes** |
| UVC_XPCS_4HR_DN | 0.00 | 130 |
| UVB_NHEK1_DN | 0.00 | 138 |
| HSA04010_MAPK_SIGNALING_PATHWAY | 0.10 | 95 |
| HSA04510_FOCAL_ADHESION | 0.41 | 99 |
| CTNNB1_oncogenic_signature | 0.48 | 32 |
| HSA04350_TGF_BETA_SIGNALING_PATHWAY | 0.73 | 37 |
| TRANSCRIPTION_ACTIVATOR_ACTIVITY | 1.70 | 63 |
| HSA04810_REGULATION_OF_ACTIN_CYTOSKELEMT_DN | 1.98 | 28 |
| HSA04540_GAP_JUNCTION | 2.06 | 43 |
| HSA04370_VEGF_SIGNALING_PATHWAY | 2.13 | 89 |
| HSA04310_WNT_SIGNALING_PATHWAY | 2.17 | 28 |
| EMT_DN | 2.72 | 27 |
| HSA04340_HEDGEHOG_SIGNALING_PATHWAY | 2.81 | 30 |
| BRCA1_OVEREXP_DN | 3.68 | 53 |
| BASSO_GERMINAL_CENTER_CD40_DN | 4.40 | 53 |
