# Supplementary material for: Gene expression in tumor cells and stroma in dsRed 4T1 tumors in eGFP-expressing mice with and without enhanced oxygenation
Source: BMC Cancer. 2012 Jan 17;12:21. doi: 10.1186/1471-2407-12-21 (PMC3274430; doi:10.1186/1471-2407-12-21)
Supplement: Additional file 3 — Table S2. Cellular processes, pathways and molecular function. Gene set enrichment analysis (GSEA) after daily hyperbaric oxygen (HBO) treatment of tumor cells. [file 1471-2407-12-21-S3.PDF]

Table S2: Cellular processes, pathways and molecular function. Gene set enrichment analysis (GSEA) after daily hyperbaric oxygen (HBO) treatment of tumor cells.

**Induced_daily HBO treatment**	**FDR**	**No.Genes**
HSA01430_CELL_COMMUNICATION	0.00	26
HSA00190_OXIDATIVE_PHOSPHORYLATION	0.00	53
ELECTRON_TRANSPORT_CHAIN	0.00	49
EMT_DN	0.14	31
SERUM_FIBROBLAST_CELLCYCLE	0.38	38
HSA04115_P53_SIGNALING_PATHWAY	1.54	22
ROS_MOUSE_DN	2.99	26
HSA04512_ECM_RECEPTOR_INTERACTION	4.49	26
**Down_daily HBO treatment**	**FDR**	**No.Genes**
UVC_TTD_4HR_DN	0.00	149
UVC_XPCS_8HR_DN	0.00	172
UVC_XPCS_ALL_DN	0.00	132
UVC_HIGH_ALL_DN	0.00	196
CTNNB1_oncogenic_signature	1.00	31
HEMAOPOSIS_RELATED_TRANSCRIPTION_FACTORS	1.49	29
SHEPARD_POS_REG_OF_CELL_PROLIFERATION	1.49	24
HSA04010_MAPK_SIGNALING_PATHWAY	2.23	75
HSA04060_CYTOKINE_CYTOKINE_RECEPTOR_INTERACTION	2.32	41
HSA04540_GAP_JUNCTION	2.38	24
BRCA1KO_MEF_DN	3.31	38
BRENTANI_TRANSCRIPTION_FACTORS	3.38	29
HSA04630_JAK_STAT_SIGNALING_PATHWAY	5.10	45
