# Supplementary material for: Gene expression in tumor cells and stroma in dsRed 4T1 tumors in eGFP-expressing mice with and without enhanced oxygenation
Source: BMC Cancer. 2012 Jan 17;12:21. doi: 10.1186/1471-2407-12-21 (PMC3274430; doi:10.1186/1471-2407-12-21)
Supplement: Additional file 4 — Table S3. Cellular processes, pathways and molecular function. Gene set enrichment analysis (GSEA) after intermittent hyperbaric oxygen (HBO) treatment of stroma cells. [file 1471-2407-12-21-S4.PDF]

Table S3:.Cellular processes, pathways and molecular function. Gene set enrichment analysis (GSEA) after intermittent hyperbaric oxygen (HBO) treatment of stroma cells.

**Induced_Intermittent HBO treatment**	**FDR**	**No.Genes**
CHEN_HOXA5_TARGETS_UP	0.00	105
BASSO_GERMINAL_CENTER_CD40_UP	0.00	41
BOQUEST_CD31PLUS_VS_CD31MINUS	0.00	130
ELECTRON_TRANSPORTER_ACTIVITY	0.00	47
HSC_INTERMEDIATEPROGENITORS_SHARED	0.00	67
VEGF_MMMEC_6HRS_UP	0.21	26
G1_TO_S_CELL_CYCLE_REACTOME	0.23	31
VEGF_MMMEC_ALL_UP	1.42	47
HDACI_COLON_CUR48HRS_UP	2.10	33
**Down_Intermittent HBO treatment**	**FDR**	**No.Genes**
HSA04660_T_CELL_RECEPTOR_SIGNALING_PATHWAY	0.00	59
HSA04630_JAK_STAT_SIGNALING_PATHWAY	0.09	62
UVC_XPCS_4HR_DN	0.10	129
UVB_NHEK1_DN	0.11	145
APOPTOSIS	0.32	37
HSA04060_CYTOKINE_CYTOKINE_RECEPTOR_INTERACTION	0.40	95
CTNNB1_oncogenic_signature	0.40	38
HSA04920_ADIPOCYTOKINE_SIGNALING_PATHWAY	0.56	38
HSA04620_TOLL_LIKE_RECEPTOR_SIGNALING_PATHWAY	1.40	51
APOPTOSIS_GENMAPP	1.70	27
MAPKPATHWAY	2.30	56
KERATINOCYTEPATHWAY	2.30	27
DNMT1_KO_UP	3.50	38
HADDAD_CD45CD7_PLUS_VS_MINUS_DN	3.84	31
CELL_SURFACE_RECEPTOR_LINKED_SIGNAL_TRANSDUCTION	3.96	63
