# Supplementary material for: Gene expression in tumor cells and stroma in dsRed 4T1 tumors in eGFP-expressing mice with and without enhanced oxygenation
Source: BMC Cancer. 2012 Jan 17;12:21. doi: 10.1186/1471-2407-12-21 (PMC3274430; doi:10.1186/1471-2407-12-21)
Supplement: Additional file 5 — Table S4. Cellular processes, pathways and molecular function. Gene set enrichment analysis (GSEA) after daily hyperbaric oxygen (HBO) treatment of stroma cells. [file 1471-2407-12-21-S5.PDF]

Table S4: Cellular processes, pathways and molecular function. Gene set enrichment analysis (GSEA) after daily hyperbaric oxygen (HBO) treatment of stroma cells.

Induced_Daily HBO treatment	FDR	No.Genes
HSA00190_OXIDATIVE_PHOSPHORYLATION	1.60	47
HSA04512_ECM_RECEPTOR_INTERACTION	2.82	27
HSA01430_CELL_COMMUNICATION	3.50	19
HSA01030_GLYCAN_STRUCTURES_BIOSYNTHESIS_1	4.30	39
HSA04510_FOCAL_ADHESION	5.49	53
**Down_Daily HBO treatment**	**FDR**	**No.Genes**
HSA04660_T_CELL_RECEPTOR_SIGNALING_PATHWAY	0.00	47
HSA04650_NATURAL_KILLER_CELL_MEDIATED_CYTOTOXICITY	0.00	37
HSA04060_CYTOKINE_CYTOKINE_RECEPTOR_INTERACTION	0.00	68
HSA04630_JAK_STAT_SIGNALING_PATHWAY	0.00	50
HSA04620_TOLL_LIKE_RECEPTOR_SIGNALING_PATHWAY	0.00	35
HSA04210_APOPTOSIS	0.03	30
HSA04640_HEMATOPOIETIC_CELL_LINEAGE	0.03	36
HSA04514_CELL_ADHESION_MOLECULES	0.14	34
HSA04010_MAPK_SIGNALING_PATHWAY	3.40	81
HSA04670_LEUKOCYTE_TRANSENDOTHELIAL_MIGRATION	4.23	34
REGULATION_OF_T_CELL_PROLIFERATION	4.95	45
